# Posterior averaging with Gaussian naive Bayes and the R package RandomGaussianNB for big-data classification

**DOI:** 10.3389/fdata.2025.1706417

**Published:** 2025-12-11

**Authors:** Patchanok Srisuradetchai

**Affiliations:** Department of Mathematics and Statistics, Faculty of Science and Technology, Thammasat University, Khlong Luang, Pathum Thani, Thailand

**Keywords:** classification, bootstrap aggregation, ensemble learning, R package, probabilistic calibration

## Abstract

RandomGaussianNB is an open-source R package implementing the posterior-averaging Gaussian naive Bayes (PAV-GNB) algorithm, a scalable ensemble extension of the classical GNB classifier. The method introduces posterior averaging to mitigate correlation bias and enhance stability in high-dimensional settings while maintaining interpretability and computational efficiency. Theoretical results establish the variance of the ensemble posterior, which decreases inversely with ensemble size, and a margin-based generalization bound that connects posterior variance with classification error. Together, these results provide a principled understanding of the bias–variance trade-off in PAV-GNB. The package delivers a fully parallel, reproducible framework for large-scale classification. Simulation studies under big-data conditions—large samples, many features, and multiple classes—show consistent accuracy, low variance, and agreement with theoretical predictions. Scalability experiments demonstrate near-linear runtime improvement with multi-core execution, and a real-world application on the Pima Indians Diabetes dataset validates PAV-GNB's reliability and computational efficiency as an interpretable, statistically grounded approach for ensemble naive Bayes classification.

## 1 Introduction

Machine learning has become a cornerstone of modern data-driven research, advancing pattern recognition, classification, and forecasting across domains such as natural language processing, healthcare, and energy systems. Deep-learning architectures like convolutional and recurrent neural networks (LeCun et al., 1998; Hochreiter and Schmidhuber, 1997) have achieved remarkable results, yet their success depends on vast data, intensive hyper-parameter tuning, and substantial computational resources. These requirements remain prohibitive in many practical settings, prompting renewed interest in lightweight algorithms that balance accuracy, interpretability, and scalability (Mahmud et al., 2020; Wang Y. et al., 2020; Tantalaki et al., 2020; Liapis et al., 2023).

Among these, the Naive Bayes (NB) classifier stands out for its simplicity and robustness. Despite its conditional-independence assumption, NB achieves competitive performance in text analytics, bioinformatics, and medical diagnostics (Rish, 2001; Jurafsky and Martin, 2008; Wang et al., 2007). Decades of work have refined NB through improved probability estimation, semi-parametric treatment of continuous features, and Bayesian-network generalizations (Domingos and Pazzani, 1997; Zhang H., 2004; Zhang T., 2004; Rennie et al., 2003). Empirical studies show that the Gaussian Naive Bayes (GNB) variant often performs well even when assumptions are violated, as feature-wise errors can offset one another (Hand and Yu, 2001; Soria et al., 2011). However, real-world data frequently deviate from Gaussianity, exhibiting skewness or heavy tails that reduce accuracy (Boulle, 2007).

To address these issues, researchers have developed distribution-aware extensions such as alpha-skew GNB (Ara and Louzada, 2022) and t- or stable-distribution NB models that improve robustness to asymmetry and outliers (Bhamre et al., 2025). Ensemble-based variants, including randomized-feature and bootstrap-enhanced NB, enhance predictive stability without adding complexity (Phatcharathada and Srisuradetchai, 2025). These exploit stochastic diversity among resampled datasets and feature subsets to jointly reduce bias and variance—a principle central to the present work.

A further limitation of GNB is its conditional-independence assumption. Copula-based formulations relax this constraint by modeling dependencies among marginals, improving accuracy under correlated features (Elidan, 2012; Wang B. et al., 2020). Non-parametric variants, which replace Gaussian likelihoods with kernel-density estimators, flexibly capture arbitrary feature distributions (Boli and Wei, 2024; Shawe-Taylor and Cristianini, 2004). Together, these advances show that enriching NB with randomization, dependency modeling, or non-parametric estimation enhances generalization and calibration.

Ensemble methods such as bagging (Breiman, 1996), boosting (Schapire, 2003; Freund and Schapire, 1997), random subspaces (Ho, 1998), and random forests (Breiman, 2001) demonstrate how aggregating weak learners mitigates variance and bias (Polikar, 2006; Zhou, 2012; Dietterich and Kong, 1995). Building on these principles, Phatcharathada and Srisuradetchai (2025) proposed the randomized feature–bootstrapped naive Bayes (RFB-NB) algorithm, combining bootstrap aggregation and random feature selection to reduce correlation bias and improve calibration. Although that study established the empirical feasibility of the approach, it lacked formal theoretical analysis, generalization proofs, and a reproducible parallel implementation.

We address these gaps by developing the posterior-averaging Gaussian naive Bayes (PAV-GNB) algorithm and its open-source RandomGaussianNB R package. This study formalizes both the theoretical and algorithmic foundations of PAV-GNB, establishing its variance-reduction and generalization properties while delivering a scalable, parallel, and reproducible framework for high-dimensional classification.

The remainder of this paper is organized as follows. Section 2 reviews related work on Naive Bayes, randomized feature selection, and bootstrap ensembles. Section 3 introduces the proposed PAV-GNB algorithm and its theoretical analysis (Section 3.2), along with the benchmarking design and evaluation metrics. Section 4 presents empirical results, Section 5 details the RandomGaussianNB R package implementation, and Section 6 concludes with key findings and future directions.

## 2 Related theory and literature review

This study builds on the RFB-NB algorithm by Phatcharathada and Srisuradetchai (2025), which combines Gaussian naive Bayes (GNB) with random subspace and bootstrap aggregation. While their approach uses majority voting, we propose averaged class probabilities, where posterior probabilities are averaged across the ensemble to reduce correlation bias and improve stability. This work re-implements their framework in R, enabling reproducibility, scalability testing, and integration into R-based workflows. This section briefly reviews the theoretical foundations of GNB, randomized feature selection, and bootstrap ensemble learning, followed by a discussion of available NB software tools.

### 2.1 Gaussian naive Bayes

NB remains widely used for classification due to its interpretability, simplicity, and computational efficiency (Pearl, 1988; Bishop, 2006; Barber, 2012). The method is grounded in Bayes' theorem and assumes conditional independence of features given the class. A widely applied variant is GNB, designed for continuous-valued features. In GNB, each feature within a class is assumed to follow a Gaussian distribution. Mathematically, for a vector of features **x** = (*x*_1_, *x*_2_, …, *x*_*p*_), the posterior probability that **x** belongs to class *C*_*k*_ is given by


$$\begin{array}{l} P\left(C_{k}|\text{x}\right)=\frac{P\left(C_{k}\right)\prod_{j=1}^{p}P\left(x_{j}|C_{k}\right)}{\sum_{i=1}^{K}P\left(C_{i}\right)\prod_{j=1}^{p}P\left(x_{j}|C_{i}\right)}, \end{array}$$ (1)

where *K* denotes the number of classes and *p* the number of features (Murphy, 2012; McCallum and Nigam, 1998). The posterior probability in (1) defines the fundamental Bayesian decision rule for Gaussian naive Bayes. Under the Gaussian assumption, each conditional likelihood takes the form:


$$\begin{array}{l} P\left(x_{j}|C_{k}\right)=\frac{1}{\sqrt{2\pi \sigma_{jk}^{2}}}\exp\left(-\frac{{\left(x_{j}-\mu_{jk}\right)}^{2}}{2\sigma_{jk}^{2}}\right), \end{array}$$ (2)

where μ_*jk*_ and $\sigma_{jk}^{2}$ denote the mean and variance of feature *j* within class *C*_*k*_, respectively. Each class-conditional Gaussian likelihood in (2) specifies how continuous features contribute to posterior computation.

Despite its efficiency and practical success across tasks such as text classification and medical diagnostics, the independence assumption limits GNB's performance in real-world settings where correlations among features are common. These correlations often degrade predictive accuracy, motivating extensions that relax this assumption or combine GNB with ensemble and feature-randomization techniques (Rennie et al., 2003; Friedman et al., 1997).

### 2.2 Randomized feature selection and feature reduction methods

Feature selection and reduction methods aim to identify informative subsets of variables from the original feature space, thereby improving predictive performance, interpretability, and computational efficiency (Li et al., 2017). Formally, given an initial feature set, shown in (3).


$$\begin{array}{l} F=\left\{F_{1},F_{2},...,F_{p}\right\}, \end{array}$$ (3)

where *p* denotes the total number of available features, the objective is to find an optimal subset F^*^ ⊆ *F* that maximizes a criterion function *J*(·):


$$\begin{array}{l} F^{*}=\underset{F^{\prime }\subseteq F}{\arg\max}J\left(F^{\prime }\right), \end{array}$$ (4)

where *J*(*F*′) may represent predictive accuracy, information gain, or another performance-based criterion (Hall, 1999). The optimization criterion *J*(*F*′) in (4) captures predictive relevance or mutual information. Wrapper methods explicitly evaluate subsets through resampling or cross-validation (Guyon and Elisseeff, 2003), while filter methods employ statistical measures such as correlation or mutual information to eliminate redundant and irrelevant variables (Urbanowicz et al., 2018).

Beyond deterministic approaches, randomized feature selection introduces controlled stochasticity to diversify ensembles and mitigate feature correlations. At each iteration *t*, a random subset


$$\begin{array}{l} F_{t}=\left\{F_{t_{1}},F_{t_{2}},...,F_{t_{m}}\right\},m\ll p \end{array}$$ (5)

is drawn without replacement from the full feature set. Repeating this process across *T* iterations yields a collection of subsets {*F*_1_, *F*_2_, ..., *F*_*T*_}, each serving as the basis for training a classifier (Dietterich, 2000; Biau and Scornet, 2016; Sagi and Rokach, 2018). Randomized feature subsets in (5) diversify the ensemble, reducing correlation among base learners.

Decision-tree-guided approaches, proposed by Ratanamahatana and Gunopulos (2003), use decision tree classifiers to recursively partition the feature space, selecting subsets of features that significantly reduce classification error. Sparse selection techniques further refine this approach, as exemplified by Askari et al. (2020), who formulated sparse naive Bayes as a constrained optimization problem aiming to minimize model complexity and prediction error simultaneously.

### 2.3 Bootstrap sampling and ensemble techniques

Bootstrap sampling, first introduced by (Efron and Tibshirani 1994), is a cornerstone of modern ensemble methods, enhancing both robustness and generalizability. Given a training set


$$\begin{array}{l} D=\left\{\left(x_{1},y_{1}\right),\left(x_{2},y_{2}\right),\ldots ,\left(x_{N},y_{N}\right)\right\} \end{array}$$ (6)

where *N* is the number of samples, bootstrap sampling generates *B* resampled subsets *D*^(*b*)^ (*b* = 1, 2, ..., *B*) by sampling *N* instances with replacement from *D*. Each bootstrap subset, as shown in (6), preserves the dataset size but introduces variability, thereby enabling repeated and independent training of predictive models (Kamlangdee and Srisuradetchai, 2025; Sriprasert and Srisuradetchai, 2025).

Formally, the bootstrap subset is defined as in (7).


$$\begin{array}{l} D^{\left(b\right)}=\left\{\left(x_{i_{1}}^{\left(b\right)},y_{i_{1}}^{\left(b\right)}\right),\left(x_{i_{2}}^{\left(b\right)},y_{i_{2}}^{\left(b\right)}\right),\ldots ,\left(x_{i_{N}}^{\left(b\right)},y_{i_{N}}^{\left(b\right)}\right)\right\}. \end{array}$$ (7)

Ensemble methods such as bagging and random forests build multiple models on these bootstrap subsets, then aggregate predictions through majority voting (classification) or averaging (regression):


$$\begin{array}{l} \hat{y}\left(x\right)=\text{agg}\left\{h_{1}\left(x\right),h_{2}\left(x\right),...,h_{B}\left(x\right)\right\}, \end{array}$$ (8)

where *h*_*b*_(*x*) is the prediction from the *b*-th model, and agg(·) denotes the aggregation function. Aggregated predictions follow (8), where the ensemble output is computed via voting or averaging.

In the context of GNB, bootstrap-enhanced ensembles enable independent estimation of class-conditional distributions. For a feature *x*_*j*_ under class *C*_*k*_, the ensemble conditional probability is aggregated as shown in (9) (Mitchell, 1997; Davidson, 2004).


$$\begin{array}{l} p^{*}\left(x_{j}|C_{k}\right)=\frac{1}{B}\sum_{b=1}^{B}p\left(x_{j}|C_{k},D^{\left(b\right)}\right). \end{array}$$ (9)

The final class prediction maximizes the posterior probability aggregated across bootstrap models:


$$\begin{array}{l} C_{k}^{*}=\arg\max\left\{P\left(C_{k}\right)\prod_{j=1}^{p}p^{*}\left(x_{j}|C_{k}\right)\right\}, \end{array}$$ (10)

The final class decision rule in (10) maximizes the averaged posterior probability over all bootstrap models. This framework reduces variance, improves stability, and enhances predictive accuracy by combining multiple weak estimators.

Beyond predictive modeling, bootstrap sampling is instrumental for robust statistical inference. For instance, it enables estimation of confidence intervals for parameters of intricate statistical distributions (Panichkitkosolkul and Srisuradetchai, 2022). Given the versatility and mathematical rigor of bootstrap approaches, they remain widely adopted across various analytical contexts, from classical regression to sophisticated machine learning ensembles (DiCiccio and Efron, 1996).

### 2.4 Existing software and computational tools for NB classifiers

NB classifiers remain popular thanks to accessible, reproducible implementations in R and Python. In R, packages such as caret (Kuhn, 2008), e1071 (Meyer et al., 2024), and randomForest (Liaw and Wiener, 2002) support model training, validation, and benchmarking. Python's scikit-learn (Pedregosa et al., 2011) provides Gaussian, multinomial, and Bernoulli NB variants that integrate seamlessly into modern workflows.

Most tools rely on classical, non-ensemble NB formulations with fixed parameter estimation and limited bias–variance control. They lack mechanisms such as random feature subspace selection, bootstrap aggregation, and multi-core parallelism. Although random forests scale efficiently via bagged trees, their split-based learning differs from probabilistic NB inference, leaving current NB implementations less robust for correlated or high-dimensional data. Moreover, few R libraries include probability-calibration utilities or ensemble management beyond basic resampling, limiting users' ability to assess predictive uncertainty and computational trade-offs.

To overcome these limitations, the RandomGaussianNB R package implements the posterior-averaging Gaussian Naive Bayes algorithm within a reproducible, parallel framework. It unifies random feature selection, bootstrap aggregation, and calibration while maintaining NB interpretability. Multi-core execution via foreach (Weston and Microsoft Corporation, 2022b) and doParallel (Weston and Microsoft Corporation, 2022a) ensures scalability, providing the foundation for the algorithmic and theoretical developments detailed in Section 3.

## 3 Proposed algorithm and theoretical properties

This section outlines the posterior-averaging Gaussian Naive Bayes (PAV-GNB) algorithm and its theoretical properties—variance reduction and generalization behavior—and presents computational experiments evaluating runtime, scalability, and predictive accuracy under reproducible synthetic-data conditions.

### 3.1 Detailed algorithmic description

The RandomGaussianNB classifier implements the PAV-GNB ensemble through a fully parallel R implementation. The algorithmic steps of the proposed PAV-GNB are outlined below.

Let $D={\left\{\left({\text{x}}_{i},y_{i}\right)\right\}}_{i=1}^{n}={\left\{\left({\text{x}}_{i},C_{i}\right)\right\}}_{i=1}^{n}$ denote a training dataset with *p* predictors and *K* classes. At each ensemble iteration *t* = 1, …, *T*:

I. Bootstrap resampling (rows)
  Draw a sample *D*^(*t*)^ of size *n* with replacement from *D*.
  Sampling is stratified by class to preserve class proportions.
II. Random feature subset selection (columns)
  Select without replacement a subset of features *F*^(*t*)^ ⊂ {1, 2, ..., *p*} of size *m* = ⌊α*p*⌋, where α ∈ (0, 1] is the feature fraction.
III. Base learner training (GNB)
  For each class *k* ∈ {1, …, *K*} and feature *j* ∈ *F*^(*t*)^, estimate the Gaussian parameters
  $\mu_{kj}^{\left(t\right)}=\frac{1}{n_{k}^{\left(t\right)}}\sum_{i:y_{i}=k}x_{ij},\;{\left(\sigma_{kj}^{\left(t\right)}\right)}^{2}=\frac{1}{n_{k}^{\left(t\right)}-1}\sum_{i:y_{i}=k}{\left(x_{ij}-\mu_{kj}^{\left(t\right)}\;\right)}^{2}.$ The class prior is $\pi_{k}^{\left(t\right)}=n_{k}/n.$
IV. Prediction on new data
  For a new observation **x**, the *t*-th model computes log-posterior scores
  $$\begin{array}{l} \;\log P^{\left(t\right)}\left(C_{k}|\text{x}\right)\propto \log\pi_{k}^{\left(t\right)}\\ +\sum_{j\in F^{\left(t\right)}}\log\left[\frac{1}{\sqrt{2\pi {\left(\sigma_{kj}^{\left(t\right)}\right)}^{2}}}\exp\left(\frac{-{\left(x_{j}-\mu_{kj}^{\left(t\right)}\right)}^{2}}{2{\left(\sigma_{kj}^{\left(t\right)}\right)}^{2}}\right)\;\right]. \end{array}$$
V. Stable aggregation across models
  To avoid numerical underflow, log-posterior matrices are normalized via the log-sum-exp trick before exponentiation. Averaged class probabilities are then computed as
  $$\begin{array}{l} {\bar{P}}_{T}\left(C_{k}|\text{x}\right)=\frac{1}{T}\sum_{t=1}^{T}P_{t}\left(C_{k}|\text{x}\;\right) \end{array}$$
  and the predicted label is $Ĉ\left(\text{x}\right)=\arg\max_{k}\bar{P}\left(C_{k}|\text{x}\;\right)$.
VI. Parallel execution
  Ensemble iterations *t* are distributed across CPU cores using foreach + doParallel (Weston and Microsoft Corporation, 2022b,a). Each worker independently executes Steps I–V and returns posterior matrices for averaging.

**Figure 1:**
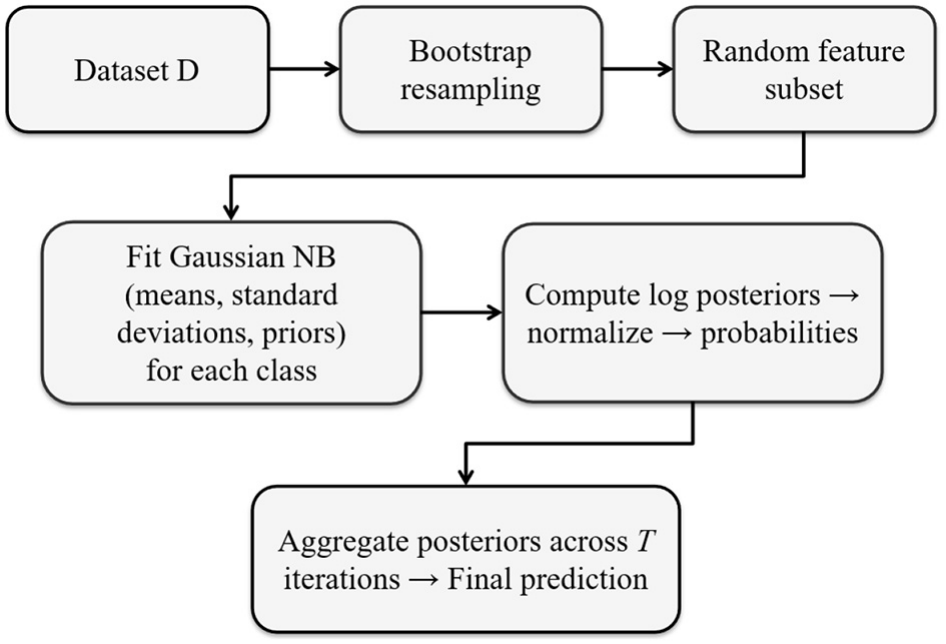
Workflow of RandomGaussianNB.

This framework combines the low-bias property of GNB with the variance-reduction effects of bootstrap aggregation and the decorrelation benefit of random subspaces. The final model object stores: $n_iter, $feature_fraction, $cores, $models, $classes, $X_train, and $y_train, accessible via standard S3 methods [print(), summary(), predict()].Figure 1 illustrates the overall workflow of the RandomGaussianNB algorithm, summarizing the key steps of stratified bootstrap resampling, random feature selection, GNB fitting, and posterior aggregation across ensemble iterations.

The following section formalizes its posterior-averaging properties and derives the generalization bound.

### 3.2 Theoretical properties of PAV-GNB

The posterior averaging Gaussian naive Bayes (PAV-GNB) ensemble aggregates the posterior probabilities from *T* independently randomized GNB classifiers trained on bootstrap samples and feature-subset draws.

Let *P*_*t*_(*C*_*k*_|**x**) denote the posterior for class *C*_*k*_ from the *t*- th model, and define the ensemble posterior


$$\begin{array}{l} {\bar{P}}_{T}\left(C_{k}|\text{x}\right)=\frac{1}{T}\sum_{t=1}^{T}P_{t}\left(C_{k}|\text{x}\right). \end{array}$$ (11)

Expectations and variances below are taken over bootstrap and feature randomization.

#### 3.2.1 Consistency and variance

Under correct model specification and regularity of the plug-in estimators, each *P*_*t*_(*C*_*k*_|**x**) is consistent for the true posterior $p^{*}\left(C_{k}|\text{x}\right)$; hence ${\bar{P}}_{T}$ is also consistent as *n* → ∞ (fixed *T*). Assuming equal marginal variance σ^2^(**x**) and average pairwise correlation ρ(**x**) across {*P*_*t*_},


$$\begin{array}{l} Var\left[{\bar{P}}_{T}\left(C_{k}|\text{x}\right)\right]=\frac{\sigma^{2}\left(\text{x}\right)}{T}\left(1+\rho \left(\text{x}\right)\left(T-1\right)\right). \end{array}$$ (12)

Smaller overlap among *D*^(*t*)^ and *F*^(*t*)^ decreases ρ(**x**), yielding near−1/*T* variance reduction.

#### 3.2.2 Quadratic-loss aggregation

For the Brier score (Brier, 1950; Geman et al., 1992; Breiman, 1996), the population minimizer is p^*^(·|**x**). Among convex mixtures of {*P*_*t*_}, the optimal weights depend on the error covariance; equal weights are optimal under exchangeability with equal variance and covariance, making simple averaging a stable, statistically efficient rule when base learners are approximately homogeneous and weakly correlated.

#### 3.2.3 Margin-based generalization bound

Let the Bayes margin be $\gamma \left(\text{X}\right)=p^{*}\left(Y|X\right)-\max_{k\neq Y}p^{*}\left(C_{k}|\text{X}\right)>0$. Suppose *E*[*P*_*t*_(*Y*|**X**)]=p^*^(*Y*|**X**) + *b*(**X**) and $Var\left[P_{t}\left(Y|\text{X}\right)\right]=\sigma^{2}\left(\text{X}\right)$ with average pairwise correlation ρ(**X**). Then, for[*u*]_+_ = max(*u*, 0),


$$\begin{array}{l} \mathrm{Pr}\left[\hat{C}\left(\text{X}\right)\neq Y\right]\leq \frac{\sigma^{2}\left(\text{X}\right)\left\{1+\rho \left(\text{X}\right)\left(T-1\right)\right\}}{T{\left[\frac{\gamma \left(\text{X}\right)}{2}-|b\left(\text{X}\right)|\right]}_{+}^{2}}, \end{array}$$ (13)

where $\hat{C}\left(\text{X}\right)=\arg\max_{k}{\bar{P}}_{T}\left(C_{k}|\text{X}\right)$. The bound decays with larger *T* and smaller ρ, and tightens with larger margin and smaller bias.

A proof sketch for (12) and (13) is provided in Appendix A, detailing the equicorrelation calculation and the Cantelli step used in the margin-based bound.

### 3.3 RandomGaussianNB simulation design and dataset generation

Synthetic datasets are generated to systematically control key characteristics, including sample size *n*, number of features *p*, number of classes *K*, and correlation structures among features. Each dataset *D* in (14) consists of feature vectors **x**_*i*_ paired with corresponding class labels *y*_*i*_:


$$\begin{array}{l} D={\left\{\left({\text{x}}_{i},y_{i}\right)\right\}}_{i=1}^{n},\;{\text{x}}_{i}\in {\mathbb{R}}^{p},\;y_{i}\in \left\{0,1,...,K-1\right\}. \end{array}$$ (14)

Each **x**_*i*_ for class *k* is sampled from a class-specific multivariate Gaussian distribution:


$$\begin{array}{l} {\text{x}}_{i}|\left(y_{i}=k\right)\sim N\left(\mu_{k},\Sigma_{k}\right),\;k=0,1,...,K-1, \end{array}$$ (15)

with class-specific mean vectors **μ**_*k*_ and covariance matrices **Σ**_*k*_.

Class overlap is regulated by adjusting **μ**_*k*_ and **Σ**_*k*_. Separability is characterized using Mahalanobis distances between class means and controlled correlation parameters. Following Schober et al. (2018), correlation thresholds are interpreted as minimal (ρ = 0.1), moderate (ρ = 0.5), and strong (ρ = 0.9) associations, while the identity structure (ρ = 0) reflects independence. The systematically defined scenarios are summarized in Table 1.

**Table 1:** Summary of class overlap levels and corresponding covariance structures.

**Overlap level**	**Mahalanobis distance ||**μ**_*i*_ − **μ**_*j*_||**	**Correlation ρ**	**Covariance structure **Σ**_*k*_**
No overlap	5	0	Identity matrix (**I**_*p*_); independent features
Low overlap	3.5	0.1	$\left(1-\rho \right)I_{p}+\rho 1_{p}1_{p}^{T}$; minimal correlation
Moderate overlap	2	0.5	$\left(1-\rho \right)I_{p}+\rho 1_{p}1_{p}^{T}$; moderate correlation
High Overlap	1	0.9	$\left(1-\rho \right)I_{p}+\rho 1_{p}1_{p}^{T}$; strong correlation

### 3.4 Computational environment

All benchmarking experiments are conducted in a standardized computing environment to ensure reproducibility:

- Processor: Intel(R) Core(TM) i9-13980HX CPU, 2.20 GHz, 24 cores (32 logical processors)
- RAM: 64 GB
- Operating System: Microsoft Windows 11 Pro (Version 10.0.26100)
- R Version: 4.4.0

Parallel computation is implemented using the parallel (R Core Team, 2025) and foreach (Weston and Microsoft Corporation, 2022b) packages, enabling performance evaluation across different core settings (1, 2, 4, 8, 16, and 24 cores).

### 3.5 Benchmarking procedure

A comprehensive benchmarking protocol was designed to evaluate the runtime, scalability, and stability of the proposed PAV-GNB classifier. Experimental scenarios of varying complexity ensure broad coverage of practical conditions and reproducibility. Each scenario was independently repeated *R* = 10 times. Statistical differences were assessed by two-way ANOVA (α = 0.05) followed by Tukey's HSD *post-hoc* tests (Tukey, 1949).

#### 3.5.1 Baseline experiments

Baseline experiments established reference performance under controlled conditions. Simulations used a fixed dataset size *n* = 1,000, dimensionality *p* = 50, three-class classification *K* = 3, feature fraction *f* = 0.25, number of iterations *T* = 100, and single-core computation (*c* = 1). Four class-overlap levels—no overlap (||μ_*i*_ − μ_*j*_|| ≈ 5, ρ = 0), low overlap (≈ 3.5, ρ = 0.1), moderate overlap (≈ 2, ρ = 0.5), and high overlap (≈ 1, ρ = 0.9)— quantified the influence of data separability on accuracy and runtime.

#### 3.5.2 Scalability analysis

To examine efficiency gains from parallel computation, moderate complexity parameters (*n* = 5000, *p* = 100, *K* = 3, *f* = 0.25, *T* = 100, ρ = 0.5) were retained while varying CPU cores *c* ∈ {1, 2, 4, 8, 16}. This analysis quantified runtime reduction and scalability efficiency *E*_*s*_ under increasing parallel resources.

#### 3.5.3 Dimensionality and sample size stress test

Robustness under high-dimensional and large-sample conditions was assessed by systematically increasing dataset size *n* ∈ {500, 1000, 5000, 10, 000, 20, 000, 50, 000, 100, 000} and dimensionality *p* ∈ {10, 50, 100, 500, 1000, 2000}, while holding *K* = 3, *f* = 0.25, *c* = 4, *T* = 100, ρ = 0.5. These experiments probed computational feasibility from small to extremely large analytic scales.

#### 3.5.4 Feature fraction sensitivity analysis

The effect of randomized feature selection was investigated by varying the feature fraction $f\in \left\{\sqrt{p}/p,1/10,1/5,1/4,1/3,1/2,1\right\},$ keeping other parameters fixed (*n* = 5000, *p* = 100, *K* = 3, *c* = 4, *T* = 100, ρ = 0.5). This clarified how subsampling proportions influence accuracy and runtime.

#### 3.5.5 High complexity and multiclass performance

Classifier effectiveness under realistic large-scale conditions was evaluated with *n* = 100, 000, *p* = 1000, *c* = 8, *f* = 0.25), and class counts *K* ∈ {2, 3, 5, 10}. Experiments were conducted under moderate (ρ = 0.5) and high (ρ = 0.9) overlap to test robustness against severe class correlation.

#### 3.5.6 Iterations and stability analysis

Finally, predictive stability was examined by varying the number of bootstrap iterations *T* = {10, 50, 100, 300, 500} while maintaining moderate complexity settings (*n* = 5000, *p* = 100, *K* = 3, *c* = 4, *f* = 0.25, ρ = 0.5). This assessed consistency of accuracy and runtime across repeated resampling.

### 3.6 Performance evaluation criteria

Three complementary metrics are used to assess computational efficiency and predictive performance:

(1) Computational Runtime (seconds): The mean total time for training and prediction across *R* independent repetitions is defined in (16):
  $$\begin{array}{l} \text{Runtime}=\frac{1}{R}\sum_{r=1}^{R}\left(T_{\text{train},r}+T_{\text{predict},r}\right), \end{array}$$ (16)
  where *T*_train, *r*_ and *T*_predict, *r*_ denote the training and prediction times of the *r-*th run.
(2) Scalability Efficiency (*E*_*S*_): Parallel-processing gains are quantified using (17) (Schryen, 2022):
  $$\begin{array}{l} {\text{E}}_{S}=\frac{T_{1}}{cT_{c}}, \end{array}$$ (17)
  where *T*_1_ is the single-core runtime, *T*_*c*_ is runtime using *c* cores, and larger values indicate better parallel efficiency.
(3) Predictive Accuracy: Classification accuracy is computed as (18) (Powers, 2011; Kummaraka and Srisuradetchai, 2025):
  $$\begin{array}{l} \text{Accuracy}=\frac{1}{n}\sum_{i=1}^{n}I\left({\hat{y}}_{i}=y_{i}\right), \end{array}$$ (18)
  where ŷ_*i*_ is the predicted class, the true class, and *I*(·) an indicator function.

## 4 Experimental results

### 4.1 Baseline performance evaluation

**Figure 2:**
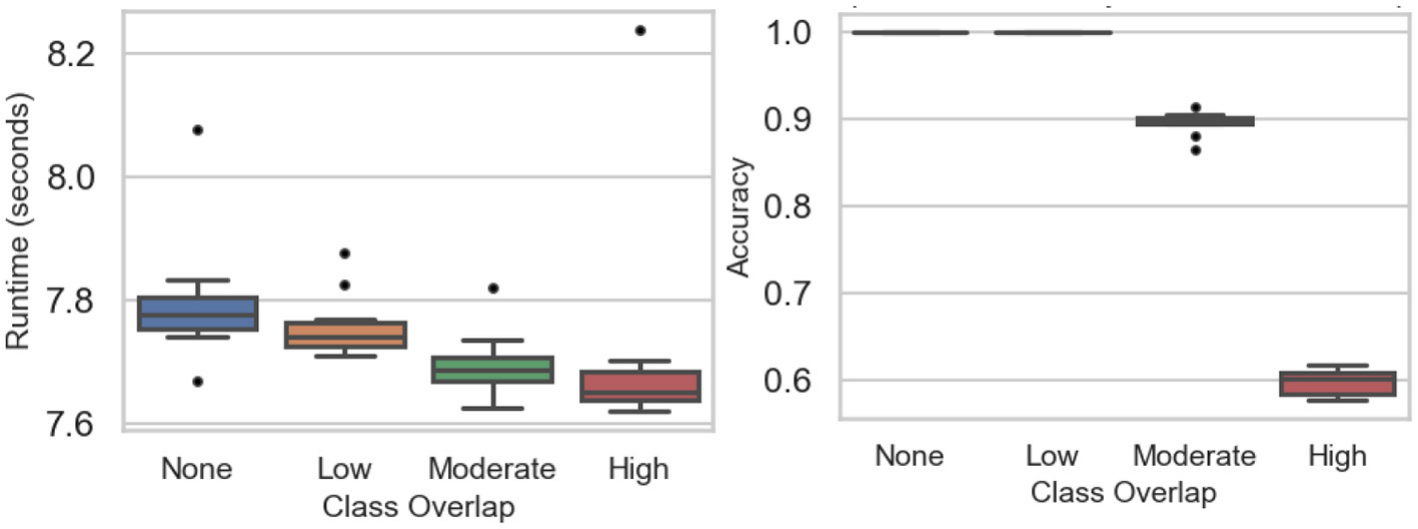
Baseline experiments: runtime **(Left)** and accuracy **(Right)** across class overlap scenarios.

Table 2 and Figure 2 summarize the baseline experiments assessing runtime and accuracy across four levels of class overlap: none, low, moderate, and high. Mean runtime remained stable as overlap increased, ranging from 7.769 s to 7.79 s. A Tukey HSD test confirmed no significant differences among these scenarios (*p*-value = 0.195), indicating that runtime was largely unaffected by class separability. Minor outliers observed in Figure 2 reflect normal variability from random initialization or hardware fluctuations rather than methodological effects.

**Table 2:** Summary of baseline experiment results.

**Overlap level**	**Runtime (seconds)**		**Accuracy (proportion)**	
	**Mean**	**Standard deviation**	**Mean**	**Standard deviation**
None	7.799	0.107	1	0
Low	7.760	0.053	1	0
Moderate	7.697	0.053	0.8963	0.0136
High	7.715	0.186	0.5974	0.0144

Accuracy, in contrast, was strongly influenced by overlap. Perfect classification (mean = 1.0) was achieved when classes were well-separated (none or low overlap). Accuracy declined to 0.896 (SD = 0.014) under moderate overlap and dropped further to 0.598 (SD = 0.014) when classes substantially intersected. These results highlight that while computational cost is stable, prediction reliability decreases sharply as class distributions become less distinct, underscoring the challenge of learning in highly overlapping settings.

### 4.2 Scalability analysis

**Figure 3:**
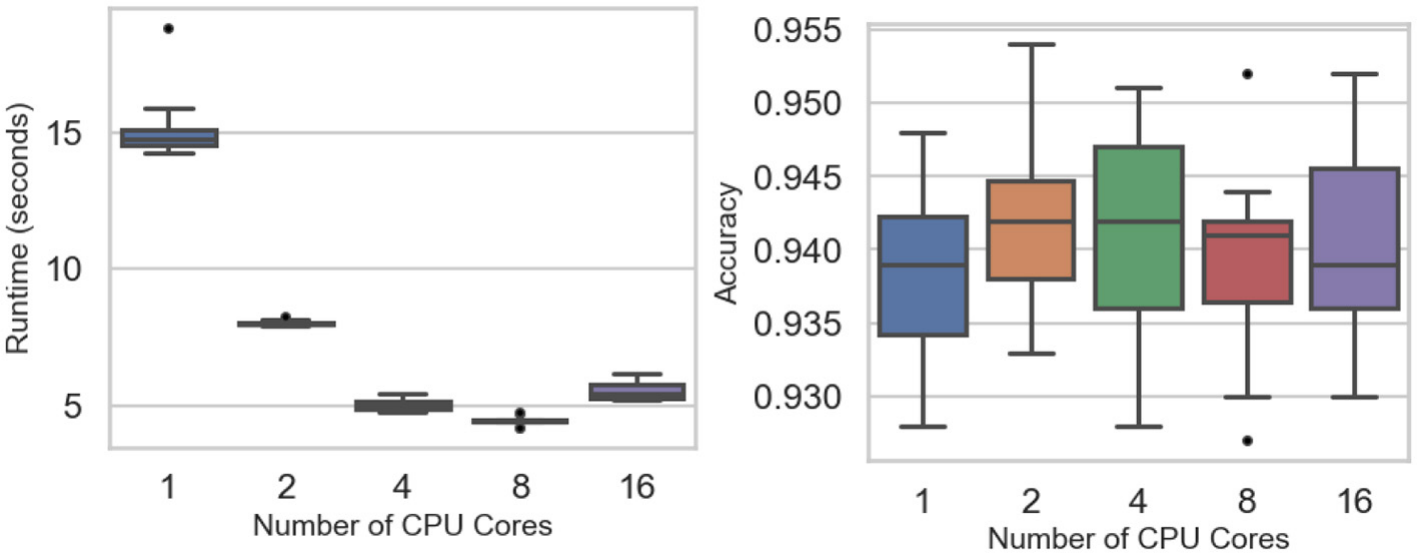
Scalability experiments: runtime **(Left)** and accuracy **(Right)** across the number of CPU cores.

Scalability experiments evaluate how computational runtime and predictive accuracy vary with the number of CPU cores. Figure 3 (left) shows a sharp runtime reduction as parallelization increases, falling from a single-core mean of 15.20 s to 8.03 s (two cores), 5.04 s (four cores), and a minimum of 4.47 s at eight cores (see Table 3). A slight rise to 5.55 s at sixteen cores suggests diminishing returns or overhead from heavy parallel execution. Two-way ANOVA confirms significant runtime differences across core settings (*F* value = 486.1, *p-*value < 0.0001), while Tukey tests find no significant gap between four and eight cores (*p-*value = 0.295) or between four and sixteen cores (*p-*value = 0.378).

**Table 3:** Summary of scalability analysis.

**Number of CPU cores**	**Runtime (seconds)**		**Accuracy (proportion)**		**Scalability efficiency**
	**Mean**	**Standard deviation**	**Mean**	**Standard deviation**	
1	15.2039	1.3474	0.9378	0.0066	1.0000
2	8.0292	0.1054	0.9422	0.0059	0.9468
4	5.0352	0.2054	0.9409	0.0081	0.7549
8	4.4742	0.1858	0.9393	0.0071	0.4248
16	5.5508	0.3407	0.9404	0.0071	0.1712

Classification accuracy remains stable across all levels of parallelism (Figure 3, right), fluctuating narrowly between 0.938 and 0.942 (Table 3). The F-test (*p-*value = 0.915) indicates no significant accuracy difference among core counts. Scalability efficiency, *E*_*s*_ = *T*_1_/(*cT*_*c*_), declines from 0.95 (two cores) to 0.75 (four cores), 0.42 (eight cores), and 0.17 (sixteen cores), confirming that parallel speedup plateaus as core usage grows.

These findings indicate that speedup improves substantially up to eight cores, beyond which performance gains taper due to synchronization overhead and data-distribution costs, consistent with typical behavior in ensemble parallelization.

### 4.3 Dimensionality and sample size stress test

#### 4.3.1 Impact of dimensionality on classifier performance

**Figure 4:**
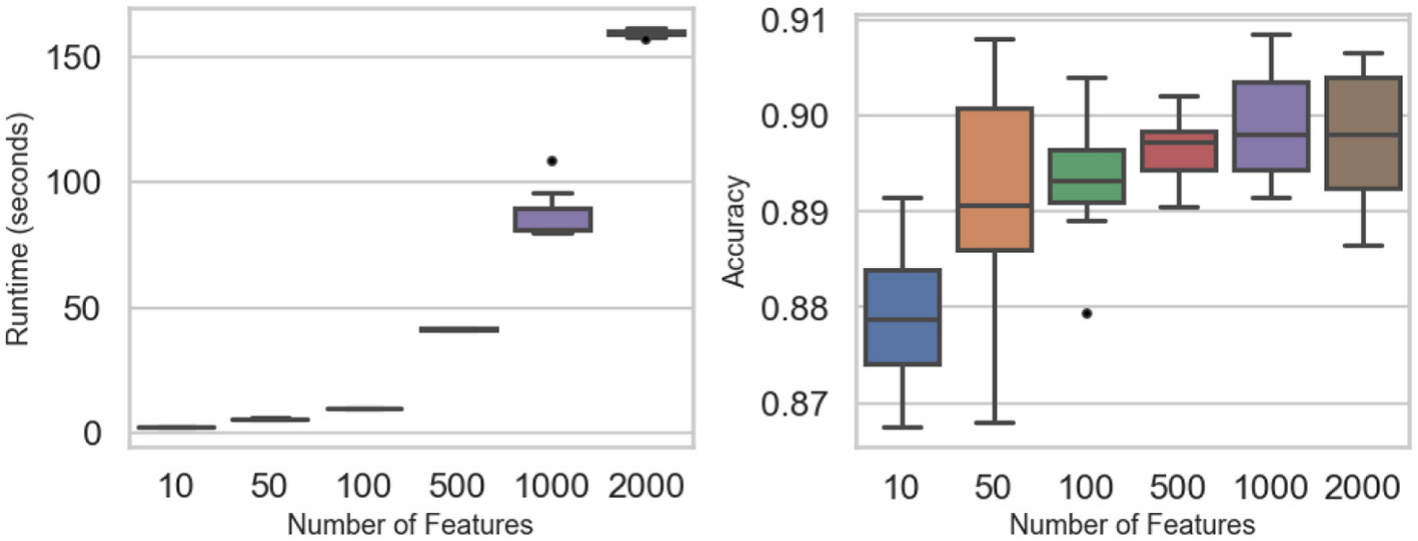
Dimensionality experiments: runtime **(Left)** and accuracy **(Right)** across the number of features.

Figure 4 and Table 4 summarize how feature dimensionality influences runtime and accuracy. Computational cost rises sharply with higher dimensions: mean runtime stays below 10 s for 10–100 features but climbs to roughly 41 s at 500, 87 s at 1,000, and 160 s at 2,000 features. Two-way ANOVA confirms significant runtime differences (*F* value = 2,418, *p*-value < 0.0001). Tukey *post-hoc* tests show no significant gap between 10 and 50 features (*p*-value = 0.475) or between 50 and 100 (*p*-value = 0.202), but all larger jumps are significant, reflecting the quadratic growth in matrix operations.

**Table 4:** Summary of dimensionality performance.

**Number of features**	**Runtime (seconds)**		**Accuracy (proportion)**	
	**Mean**	**Standard deviation**	**Mean**	**Standard deviation**
10	2.5065	0.0427	0.8794	0.0073
50	5.7172	0.1656	0.8917	0.0116
100	9.8660	0.1434	0.8934	0.0065
500	41.4558	0.3341	0.8966	0.0032
1,000	86.5477	9.6689	0.8991	0.0063
2,000	159.6616	1.3790	0.8974	0.0077

Despite these steep runtime increases, classification accuracy remains stable across all dimensional settings. Mean accuracy fluctuates only slightly—from 0.879 at 10 features to about 0.899 at 1,000 and 0.897 at 2,000—differences that are not statistically significant (*F* value = 0.562, *p*-value = 0.69). These results demonstrate that while computational demands scale rapidly with dimensionality, the predictive performance of the PAV-GNB classifier is largely unaffected, underscoring its robustness for high-dimensional data.

#### 4.3.2 Sample size stress test

**Figure 5:**
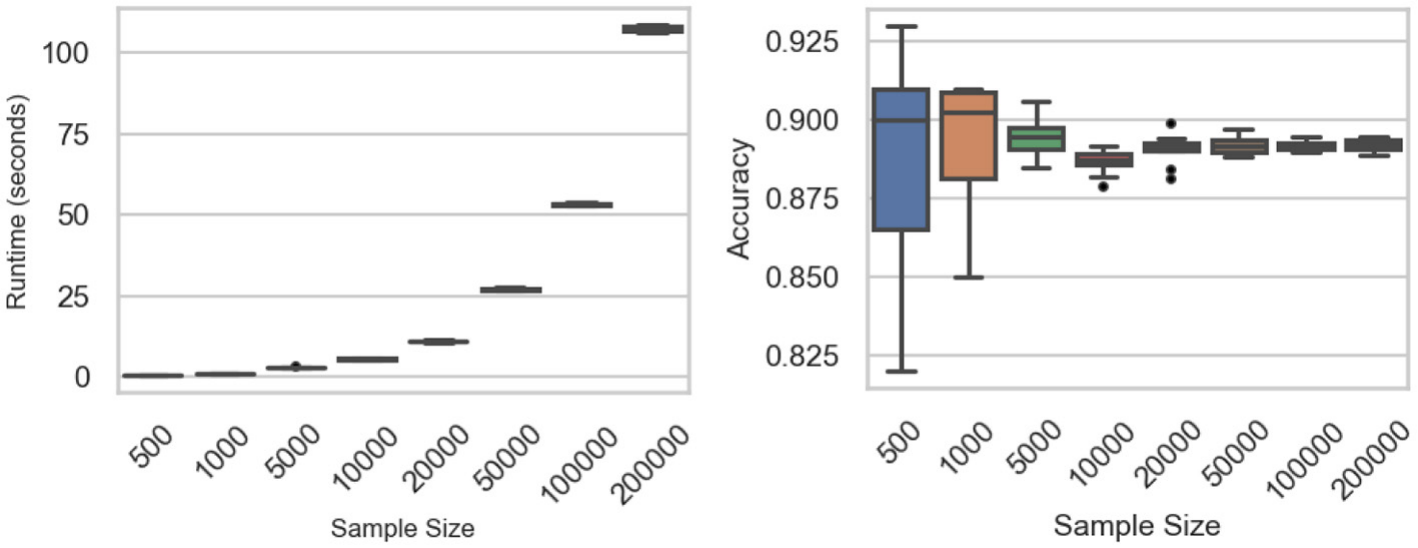
Sample size experiments: runtime **(Left)** and accuracy **(Right)** across the sample sizes.

Figure 5 and Table 5 summarize the classifier's computational performance and accuracy across sample sizes from *n* = 500 to *n* = 200,000. Runtime scales predictably with dataset size: it remains below 5 s for *n* ≤ 5,000, rises to about 10 s at *n* = 20,000, and reaches 27 s, 53 s, and 107 s for *n* = 50,000, 100,000, and 200,000, respectively. ANOVA confirms a strong effect of sample size on runtime (*F*-value = 1.05 × 10^5^, *p*-value < 0.0001), with Tukey tests showing significant pairwise differences at nearly all adjacent sizes except between 500 and 1,000 observations (*p*-value = 0.571).

**Table 5:** Summary of sample sizes stress test.

**Sample sizes**	**Runtime (seconds)**		**Accuracy (proportion)**	
	**Mean**	**Standard deviation**	**Mean**	**Standard deviation**
500	0.7959	0.0429	0.8890	0.0338
1,000	1.1024	0.0971	0.8930	0.0216
5,000	3.1095	0.1406	0.8943	0.0065
10,000	5.7346	0.1842	0.8870	0.0040
20,000	11.2272	0.2888	0.8908	0.0049
50,000	27.3318	0.2819	0.8921	0.0029
100,000	53.4161	0.3514	0.8919	0.0014
200,000	107.6518	0.8454	0.8920	0.0021

### 4.4 Feature fraction analysis

**Figure 6:**
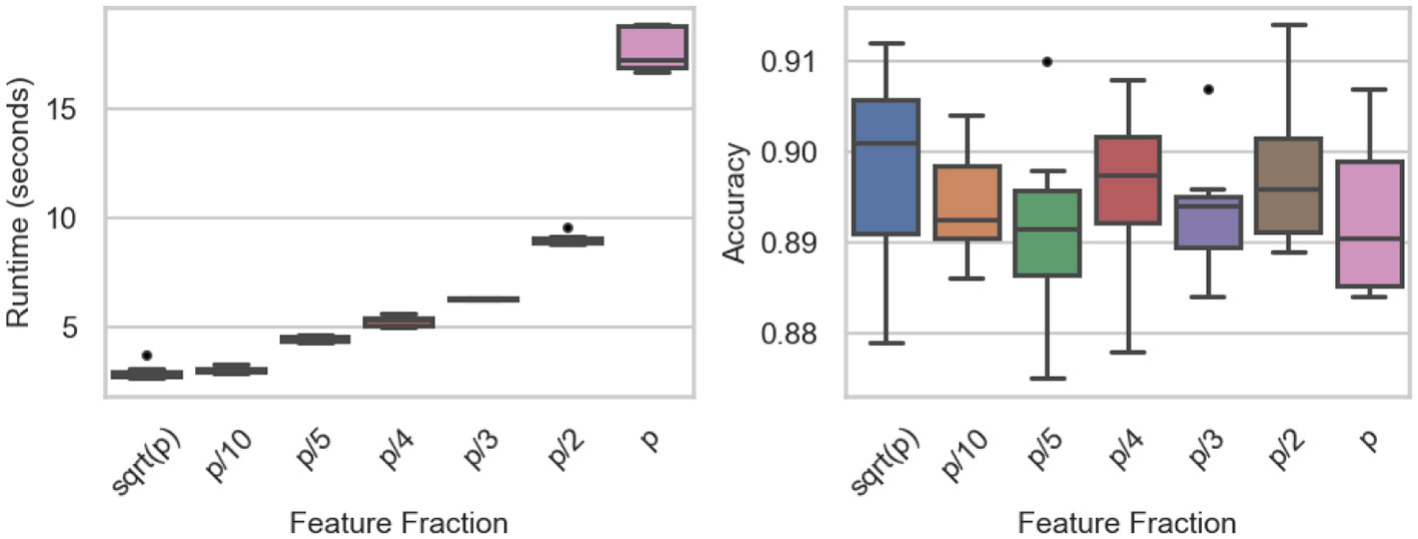
Feature fraction experiments: runtime **(Left)** and accuracy **(Right)** across the number of features.

The influence of feature subsampling on runtime and predictive accuracy is summarized in Table 6 and visualized in Figure 6. Runtime rises steadily as the fraction of utilized features increases. When small subsets such as $\sqrt{p}$ or *p*/10 are selected, mean runtime remains below 3 s, whereas employing all features (*p*/*p*) drives the mean to roughly 17.7 s. ANOVA confirms significant differences among fractions (*F*-value = 1620.89, *p*-value < 0.0001), and Tukey's tests identify strong contrasts between extreme settings (e.g., *p* vs. *p*/10, *p*/2 vs. *p*/10; both *p*-value < 0.0001). Only the comparison between $\sqrt{p}$ and *p*/10 shows no significant gap (*p*-value = 0.9963).

**Table 6:** Summary of feature fraction analysis.

**Feature fraction**	**Runtime (seconds)**		**Accuracy (proportion)**	
	**Mean**	**Standard deviation**	**Mean**	**Standard deviation**
sqrt(*p*)	2.9293	0.2974	0.8983	0.0103
*p*/10	3.0401	0.1509	0.8943	0.0057
*p*/2	9.0412	0.2266	0.8979	0.0090
*p*/3	6.2995	0.0340	0.8931	0.0064
*p*/4	5.2933	0.2344	0.8965	0.0091
*p*/5	4.4489	0.1391	0.8916	0.0093
*p*	17.7344	0.9617	0.8927	0.0082

Predictive accuracy, by contrast, remains stable across all feature fractions (Figure 6, right). Mean accuracy fluctuates slightly between 0.889 and 0.898, with ANOVA (*F* value = 0.5849, *p*-value = 0.742) and Tukey's HSD revealing no significant differences. These findings indicate that reducing the fraction of sampled features can markedly cut computation time without sacrificing classification performance, underscoring the method's efficiency under feature-randomized settings.

### 4.5 High complexity and multiclass performance analysis

**Figure 7:**
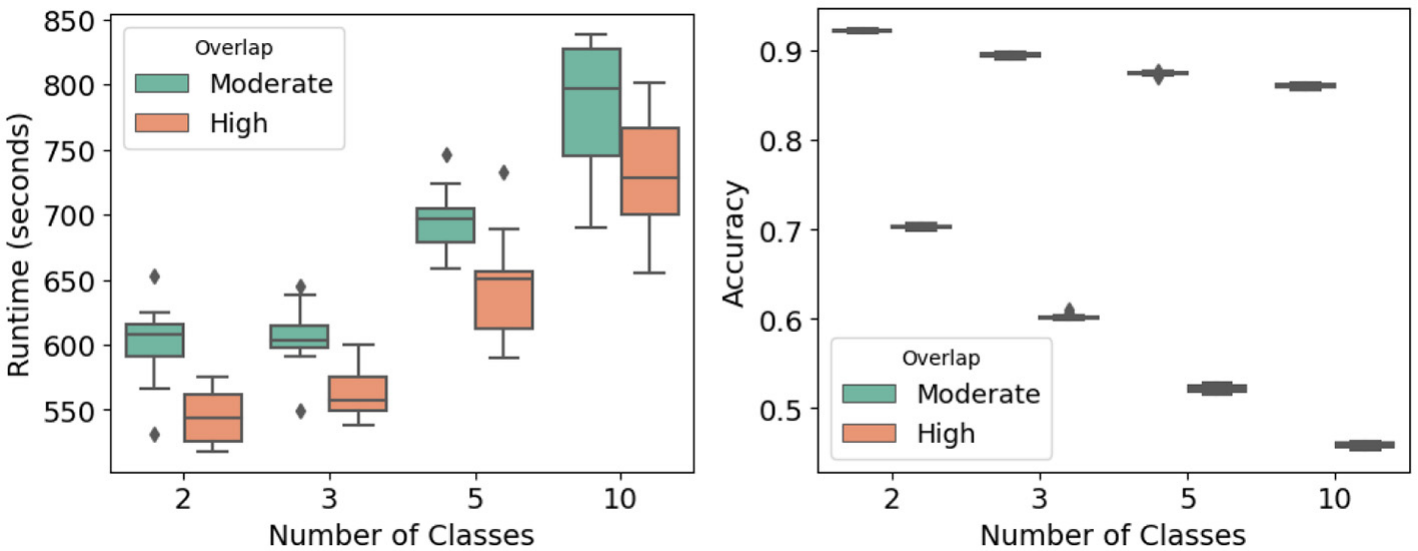
High complexity and multiclass analysis: runtime **(Left)** and accuracy **(Right)** across different numbers of classes and class overlap levels.

This experiment examines classifier behavior under demanding conditions: 100,000 samples, 1,000 features, and a feature fraction of 0.25, with the number of classes varied (*K*∈{2, 3, 5, 10}) across moderate and high overlap. As presented in Figure 7 (left) and Table 7, runtime increases with class count, from about 601 s at two classes (moderate overlap) to roughly 783 s at ten classes, while high-overlap settings yield slightly shorter runtimes at each class level. Two-way ANOVA confirms significant runtime effects of both class number and overlap (*F* value = 116.40, *p*-value < 0.0001), with Tukey's tests highlighting strong contrasts between low- and high-complexity scenarios.

**Table 7:** Summary of high complexity and multiclass analysis.

**Number of classes**	**Overlap**	**Runtime (seconds)**		**Accuracy (proportion)**	
		**Mean**	**Standard deviation**	**Mean**	**Standard deviation**
2	Moderate	601.07	0.9211	0.9211	0.0017
	High	544.67	0.7027	0.7027	0.0022
3	Moderate	605.23	0.8948	0.8947	0.0021
	High	563.53	0.6018	0.6018	0.0038
5	Moderate	696.49	0.8743	0.8743	0.0019
	High	646.59	0.5220	0.5220	0.0044
10	Moderate	783.23	0.8600	0.8599	0.0024
	High	732.94	0.4591	0.4591	0.0034

Predictive accuracy declines sharply as complexity rises. Mean accuracy reaches approximately 0.92 under the two-class moderate-overlap setting but falls to roughly 0.46 in the ten-class high-overlap case. ANOVA (*F* value = 10,304.17, *p*- value < 0.0001) and *post-hoc* comparisons verify significant accuracy reductions as overlap intensifies or the number of classes grows. These results underscore the computational and predictive challenges of simultaneous growth in class cardinality and overlap, providing a stringent stress test for the proposed PAV-GNB classifier.

### 4.6 Iterations and stability analysis

**Figure 8:**
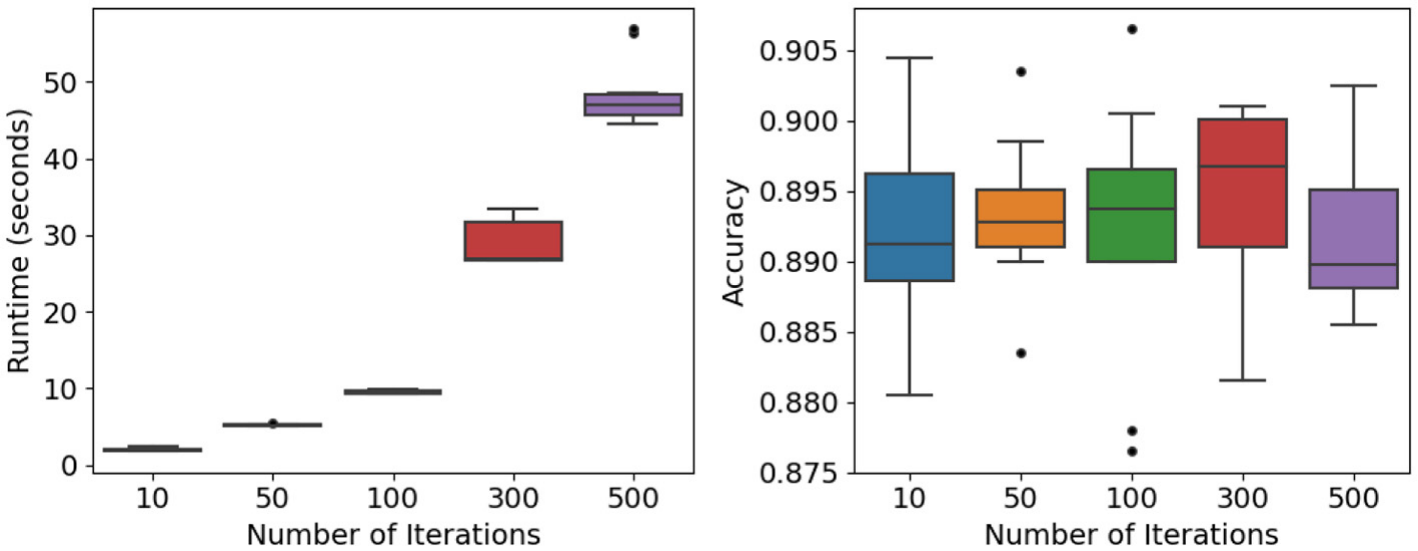
Iterations and stability analysis: runtime **(Left)** and accuracy **(Right)** across different numbers of training iterations.

The influence of training iterations on computational cost and predictive reliability was examined under moderate settings (*n* = 10, 000, *p* = 100, *K* = 3, *c* = 4, *f*= 0.25, ρ = 0.5). Iteration counts were varied across five levels (*T*∈{10, 50, 100, 300, 500}). Figure 8 (left) and Table 8 show that runtime rises nearly linearly with additional iterations—from about 2 s at *T* = 10 to roughly 48 s at *T* = 500. ANOVA confirms significant differences among these levels (*F* = 643.81, *p* < 0.0001), reflecting the expected computational burden of repeated bootstrap–feature sampling.

**Table 8:** Summary of iteration counts and stability analysis.

**Number of iterations**	**Runtime (seconds)**		**Accuracy (proportion)**	
	**Mean**	**Standard deviation**	**Mean**	**Standard deviation**
10	1.9656	0.2154	0.8924	0.0067
50	5.2237	0.0891	0.8933	0.0053
100	9.4966	0.1954	0.8922	0.0093
300	28.7804	3.0548	0.8944	0.0069
500	48.4268	4.5070	0.8919	0.0055

Despite this increase in runtime, predictive accuracy remains stable. As shown in Figure 8 (right) and Table 8, mean accuracy consistently falls within the 0.89–0.90 range, and ANOVA detects no significant variation across iteration counts (*F* value = 0.223, *p*-value = 0.924).

Conceptually, RandomGaussianNB parallels tree-based ensembles such as random forest or XGBoost (Chen and Guestrin, 2016) in using bootstrap and random subspaces for variance reduction, yet differs by aggregating probabilistic density estimates rather than split-based decisions. This preserves model interpretability while achieving comparable scalability.

## 5 Implementation and usage of the RandomGaussianNB package

### 5.1 Package overview

The RandomGaussianNB package provides a scalable and user-friendly implementation of the PAV-GNB classifier. It combines the efficiency of GNB with the robustness of bootstrap aggregation and randomized feature subsetting. The current version is maintained in a private GitHub repository; a public release and CRAN submission are planned following acceptance of this manuscript. This approach ensures future reproducibility, open access, and community support while allowing readers to cite the package as the canonical reference once it is publicly available.

### 5.2 Core functions and arguments

The main function, random_gaussian_nb(), trains an ensemble of GNB classifiers. Key arguments are:

- data: a data.frame or matrix of predictors and the response variable.
- response: the name of the response column (factor).
- n_iter: number of bootstrap iterations (default = 100).
- feature_fraction: proportion of predictors randomly selected at each iteration (default = 0.5).
- cores: number of parallel workers (default = 1).

An S3 predict() method supports both “class” (predicted label) and “prob” (posterior probability) outputs. The argument newdata allows application of the fitted model to new observations.

### 5.3 Workflow and example application

A typical workflow is illustrated using the PimaIndiansDiabetes dataset from the mlbench package (Leisch and Dimitriadou, 2024; Blake and Merz, 1998):



library(RandomGaussianNB)
library(mlbench)
# Load data
data(“PimaIndiansDiabetes”)
df <- PimaIndiansDiabetes
# Train-test split
set.seed(123)
n <- nrow(df)
train_idx <- sample(n, size = 0.7 ^*^ n)
train_df <- df[train_idx,]
test_df <- df[-train_idx,]
# Fit the PAV-GNB model
model <- random_gaussian_nb(
data = train_df,
response = “diabetes”,
n_iter = 100,
feature_fraction = 0.5,
cores = 2
)
# Predictions
probs <- predict(model,
test_df[, setdiff(names(test_df),“diabetes”)],
type = “prob”)
preds <- predict(model,
test_df[, setdiff(names(test_df),“diabetes”)],
type = “class”)
# Evaluate accuracy
mean(preds = = test_df$diabetes)


Using a 70/30 train–test split, the PAV-GNB classifier achieved an accuracy of 0.762, exceeding or matching the performance of several standard algorithms—classical GNB (0.758), k-nearest neighbors (k = 5; 0.753), support vector machine with radial kernel (0.758), random forest (0.745), decision tree (0.710), and a simple neural network (0.662) (Table 9). This experiment highlights the package's balance between simplicity, interpretability, and predictive strength relative to widely used classifiers.

**Table 9:** Accuracy comparison of PAV-GNB with other standard classifiers on the PimaIndiansDiabetes dataset.

**Models**	**Accuracy**
RandomGaussianNB	0.762
Gaussian Naive Bayes	0.758
k-NN (k = 5)	0.753
Random forest	0.745
Decision tree	0.710
SVM (Radial)	0.758
Neural network	0.662

The comparative accuracy in this Table shows that RandomGaussianNB performs competitively with widely adopted classifiers—including GNB, k-NN, SVM, and random forest—while preserving full interpretability and computational efficiency. As detailed in Section 4, its near-linear scalability under parallel execution becomes more advantageous for high-dimensional or large-sample datasets, confirming RandomGaussianNB as a practical and scalable ensemble alternative to conventional models.

## 6 Conclusions

This study presented RandomGaussianNB, an open-source R package implementing the PAV-GNB algorithm—a scalable ensemble extension of GNB integrating bootstrap resampling, randomized feature subspaces, and posterior averaging. Theoretical analysis (Section 3.2) showed that posterior averaging minimizes quadratic risk, achieves mean-square optimality among convex combinations of base posteriors, and reduces variance under correlated features. The derived margin-based generalization bound links posterior variance to classification error, formalizing the ensemble's bias–variance balance.

Algorithmically (Section 3.1), PAV-GNB stabilizes GNB through ensemble randomization and parallel computation. The RandomGaussianNB package offers a reproducible S3 implementation and benchmarking pipeline for assessing accuracy, calibration, and scalability. Simulation studies (Sections 4.1–4.6) confirmed theoretical predictions—showing consistent accuracy, low variance, and stable calibration across diverse data conditions. Parallel execution achieved near-linear runtime gains, validating computational scalability.

A real-world test on the Pima Indians Diabetes dataset verified its reliability, matching or outperforming standard classifiers (classical GNB, k-NN, decision tree, random forest, SVM) with interpretability and efficiency. Future work will extend support to multi-modal data, advanced calibration, and cross-language use, reinforcing PAV-GNB as a statistically grounded and efficient framework for large-scale probabilistic classification.

## Data Availability

Publicly available datasets were analyzed in this study. This data can be found here: https://cran.r-project.org/package=mlbench.
