# Supplementary material for: Posterior averaging with Gaussian naive Bayes and the R package RandomGaussianNB for big-data classification
Source: Front Big Data. 2025 Dec 11;8:1706417. doi: 10.3389/fdata.2025.1706417 (PMC12738300; doi:10.3389/fdata.2025.1706417)
Supplement: Supplementary file 1 [file Data_Sheet_1.docx]

# Appendix

**Variance of the ensemble posterior (Eq. 12)**

Let $Z_{t} =P_{t} (C_{k} \mid\mathbf{X})$ for $t=1,2,...,T$, and write $\bar{Z}_{T}=\sum_{t=1}^{T} Z_{T}/T$. Under the equicorrelation assumption,

$Var\left(Z_{t} \right)=\sigma^{2}\left(\mathbf{X}\right)$ and $Corr\left(Z_{2},Z_{t} \right)=\rho\left(\mathbf{X}\right)$.

Equivalently, $Cov\left(\mathbf{Z} \right)=$ $\sigma^{2}\left[\left(1-\rho\right)I_{T}+\rho\mathbf{11}^{T}\right]$. Then

$$Var\left(\bar{Z}_{T}\right)=\frac{1}{T^{2}}\mathbf{1}Cov\left(\mathbf{Z}\right)\mathbf{1}=\frac{1}{T^{2}}\left[\sigma^{2}T+\sigma^{2}T\rho\left(T-1\right)\right]$$

which gives (12). The factor $\rho$ is reduced by decreasing overlap among the bootstrap samples and feature subsets, yielding near $-1/T$ variance decay when $\rho\to0$.

**Margin-based generalization bound (Eq. 13)**

Let $Y$ be the true class and define the Bayes margin $\gamma(\mathbf{X})=p^{*}(Y\mid X)-\max_{k\neq Y} p^{*}(C_{k}\mid\mathbf{X})>0$. Write the mean–variance decomposition of a base posterior as

$$E\left(Z_{t}|\mathbf{X} \right)=p^{*}(Y\mid\mathbf{X})+b(\mathbf{X}), Var\left(Z_{t}|\mathbf{X} \right)=\sigma^{2}(\mathbf{X})$$

with average pairwise correlation $\rho(\mathbf{X})$. Misclassification of the ensemble classifier $\hat{C}(\mathbf{X})=\mathrm{argmax}_{k}\bar{P}_{T}\left(C_{k}|\mathbf{X}\right)$ occurs only if

$$\bar{Z}_{T}\leq p^{*}(Y\mid\mathbf{X})-\frac{\gamma(\mathbf{X})}{2}.$$

Set $a(\mathbf{X})=\frac{\gamma(\mathbf{X})}{2}-\left|b(\mathbf{X})\right|$ and note

$$\bar{Z}_{T}-E\left[\bar{Z}_{T}|\mathbf{X}\right]\leq-a(\mathbf{X}) \Rightarrow\Pr\left[\bar{Z}_{T}-E\left(\bar{Z}_{T}\right)\leq-a(\mathbf{X})\right].$$

By the one-sided Chebyshev (Cantelli) inequality,

$$\Pr\left[\bar{Z}_{T}-E\left(\bar{Z}_{T}\right)\leq-a(\mathbf{X})\right]\leq\frac{Var\left(\bar{Z}_{T}\right)}{Var\left(\bar{Z}_{T}\right)+a^{2}}.$$

Using $Var\left(\bar{Z}_{T}\right)$ from (12) and $a(\mathbf{X})=\left[\frac{\gamma(\mathbf{X})}{2}-\left|b(\mathbf{X})\right|\right]_{+}$ yields the exact bound

$$\Pr\left[\hat{C}(\mathbf{X})\neq Y\right]\leq\frac{\sigma^{2}(\mathbf{X})\left\{1+\rho(\mathbf{X})\left(T-1\right)\right\}}{\frac{\sigma^{2}(\mathbf{X})}{T}\left\{1+\rho(\mathbf{X})\left(T-1\right)\right\}+\left[\frac{\gamma(\mathbf{X})}{2}-\left|b(\mathbf{X})\right|\right]_{+}^{2}},$$

For a simpler (looser) display, (13) in Section 3.2 omits the $+Var\left(\bar{Z}_{T}\right)$ term in the denominator, giving

$$\Pr\left[\hat{C}(\mathbf{X})\neq Y\right]\leq\frac{\sigma^{2}(\mathbf{X})\left\{1+\rho(\mathbf{X})\left(T-1\right)\right\}}{T\left[\frac{\gamma(\mathbf{X})}{2}-\left|b(\mathbf{X})\right|\right]_{+}^{2}}.$$

This makes explicit how ensemble size $T$, correlation $\rho$, bias $|b|$, and Bayes margin $\gamma$ control the error.
